# Clinical Efficacy of Physical Factors Combined with Early Psychological Intervention in Treatment of Patients with Chronic Limb Pain

**Published:** 2019-05

**Authors:** Liang QIN, Caihong CUI, Yuling HUO, Xincai YANG, Yuqian ZHAO

**Affiliations:** 1. Department of Rehabilitation Medicine, Affiliated Hospital of Hebei University, Baoding 071000, P.R. China; 2. Department of Hepatology, Baoding Infectious Diseases Hospital, Baoding 071000, P.R. China; 3. Department of Intensive Care Unit, Baoding Infectious Diseases Hospital, Baoding 071000, P.R. China

**Keywords:** Chronic limb pain, Physical factors, Early psychological intervention, Clinical efficacy

## Abstract

**Background:**

To investigate the clinical efficacy of physical factors combined with early psychological intervention in treatment of patients with chronic limb pain.

**Methods:**

A total of 132 patients with chronic limb pain admitted to Affiliated Hospital of Hebei University, Baoding, China from June 2014 to June 2017 were enrolled. The patients were divided into control group (n=66) and observation group (n=66) according to the random number table method. Both groups of patients were treated with physical factors, and the patients in the observation group were also treated with early psychological intervention. PHQ-15 pain factor score, visual analog pain score (VAS score), Hamilton depression rating scale (HAMD) and clinical efficacy were compared between two groups.

**Results:**

Difference of PHQ-15 pain factor score, VAS score and HAMD score between two groups before treatment were not statistically significant (*P*=0.091, 0.161, 0.078). At the end of treatment and at 8 weeks of follow-up, PHQ-15 pain factor score, VAS score, and HAMD score of observation group were lower than those of control group, and the differences were statistically significant (*P*=0.045, 0.014; 0.011, 0.025; 0.030, 0.015). Total clinical effective rates of observation group and control group were 92.43% and 86.37%, respectively, and the differences were statistically significant (*P*=0.019).

**Conclusion:**

Compared with physical factors alone, combination of physical factors and early psychological intervention can significantly alleviate the pain and improve the depression of patients with chronic limb pain. It should be promoted in clinical practices.

## Introduction

Chronic limb pain is caused by aseptic inflammation or chronic strain. Chronic limb pain patients often show neck and shoulder pain, limb joint pain, swelling of the affected side and limited function. In recent years, incidence of chronic limb pain has increased year by year, and has gradually become the most common occupational disease in the world (1).

The occurrence of chronic limb pain is related to a variety of factors, including gender, age, height, dark gray economy status, weight, muscle strength, health status, smoking, posture deformity, occupation and so on (2). Chronic physical pain is also closely related to the patient’s personal psychology (3, 4). Surgical therapy, injection therapy and minimally invasive therapy are often used in clinical Chinese and Western medicine treatment. Many treatments such as massage and acupuncture are used in traditional Chinese medicine treatment. With the increase of work pressure and the accelerated pace of life, patients with chronic limb pain are often accompanied by negative emotions such as anxiety and depression, therefore, treatment with physical factors alone usually fail to provide satisfactory results (5).

Therefore, we investigated the clinical efficacy of physical factors combined with early psychological intervention in treatment of patients with chronic limb pain.

## Methods

### General information

A total of 132 patients with chronic limb pain admitted to Affiliated Hospital of Hebei University, Baoding, China from June 2014 to June 2017 were enrolled. Patients were divided into control group (n=66) and observation group according to random number table (n= 66). Among 66 patients in the observation group, 41 were male and 25 were female, and age ranged from 19 to 88 yr old, with a mean age of (44.45 ± 5.66) years. Course of disease was 6 months to 5 yr, with a mean course of (20.43±5.23) months. In control group, there were 40 males and 26 females, and age ranged from 65 to 85 yr old, with a mean age of (73.78±5.78) years. Course of disease ranged from 6 months to 5 yr, with a mean course of (21.06 ± 5.31) months. No significant differences in general data were found between 2 groups.

This research was reviewed and approved by the Ethics Committee of Affiliated Hospital of Hebei University. All patients enrolled were aware of this research and each one and/or their guardians signed an informed consent.

Inclusion criteria: 1) Patients with limb pain for more than 6 months; 2) Patients aged 18 years or older; 3) Patients who volunteered to participate in this study and have signed informed consent.
Exclusion criteria: 1) Patients with a history of psychological intervention or antidepressant treatment; 2) Patients with mental or mental abnormalities; 3) Patients took analgesic drug or with a history of alcohol dependence; 4) Patients with physical illness or brain organic disease; 5) Pregnant or lactating patients.

### Methods

Both groups of patients were treated with physical factors, and patients in the observation group were combined with early psychological intervention.

### Physical factor therapy

Patients were treated with Chinese medicine fumigation and audio electric therapy.

***Chinese medicine fumigation***: Traditional Chinese medicine fumigation multi-function treatment machine was used. In a special steamer, Chinese medicine such as safflower, angelica, scorpion, pepper, eucommia, aconite, dog ridge, etc. were mixed and 3 kg–4 kg water is added. Treatment temperature was set to 39 °C–45 °C, each treatment was performed for 30 min.

***Audio electric therapy***: audio electric therapy machine was used for treatment. Two 4 mm thick 10 cm × 5 cm electrode plates were padded with cotton and soaked in 0.9% sodium chloride solution. Two electrodes were placed in the armpit and knee and fixed with an elastic band to control the current intensity at 30–50 mA. Each treatment was performed for 20–30 min.

### Early psychological intervention treatment

1. ***Psychological diagnosis:*** Patients are encouraged to express their understanding and worries about their own pain in the form of collective discussion and group discussion. Causes of misperceptions such as depression and anxiety are analyzed.
2. ***Correcting false cognition:*** Multiple methods of exercise are used to help patients correct their original misconceptions and form reasonable cognition to eliminate or alleviate bad emotions. Specific methods: Patients who have been cured were invited to present their own opinions and debate with patients about their misconceptions, so that patients can realize that although pain cannot be completely eliminated, it will not produce terrible consequences, therefore negative emotions of anxiety and depression will be relieved.
3. Strengthen the newly formed reasonable beliefs, such as: I should learn to adapt to the pain, coexist with it, and carry out mental function training such as imaginary relaxation training and abdominal breathing to help patients form correct beliefs and ways of thinking to cope with chronic pain. 1) Imagine relaxation training: beautiful and soothing music, beautiful scenery and guiding words are used to help patients recall the feelings of pleasure, 15 – 20 min for each time, twice per week, totally 20 times. 2) Abdominal breathing training: Semi-recumbent position was taken with one hand on the chest and the other on the abdomen. After exhaling breath, inhale with the nose, and slowly exhale the gas after 3 seconds of psychological silence. Gas enters the abdomen as it inhales, feeling that the hand on the abdomen is pushed up. At the same time, let the patient imagine that stress and trouble are slowly excluded from the body with exhalation, 15 min–20 min per training session, once a day.

### Observation indicators

PHQ-15 pain factor score, VAS score, HAMD score and clinical efficacy were compared between two groups. PPHQ-15 pain factor, VAS and HAMD scoring were performed before treatment, at the end of treatment and at 8 weeks of follow-up. The results were compared. Clinical efficacy: HAMD score reduction rate was used to determine the efficacy, <25% is invalid, 25%–50% is effective, 50%–80% is markedly effective, ≥ 80% is cured.

### Statistical methods

SPSS 20.0 software (Chicago, IL, USA) was used for statistical analysis. Measurement data and count data were compared by *t* test and χ^2^ test respectively. Differences at *P*<0.05 were statistically significant.

## Results

### Comparison of PHQ-15 pain factor scores

Difference of PHQ-15 pain factor scores between two groups before treatment were not statistically significant (*P*=0.091). PHQ-15 pain factor scores of observation group were lower than those of control group at the end of treatment and 8 weeks of follow-up (*P*=0.045, 0.014) (Table 1).

**Table 1:** Comparison of PHQ-15 pain factor scores (points)

***Groups***	***Cases (n)***	***Before treatment***	***End of treatment***	***8 weeks of follow-up***
Observation	66	4.63±1.90	1.15±0.94	0.87±0.32
Control	66	4.58±1.79	2.20±1.13	1.65±0.57
*t*		1.679	2.013	2.559
*P*		0.091	0.045	0.014

### VAS score comparison

Differences of VAS scores between two groups before treatment were not statistically significant (*P*=0.161). VAS scores of observation group were lower than those of control group at the end of treatment and 8 weeks of follow-up (*P*=0.011, 0.025) (Table 2).

**Table 2:** VAS score comparison (points)

***Groups***	***Cases (n)***	***Before treatment***	***End of treatment***	***8 weeks of follow-up***
Observation	66	7.98±2.11	2.83±1.22	1.21±0.71
Control	66	8.06±2.25	4.51±1.39	2.73±1.05
*t*		1.623	2.573	2.489
*P*		0.161	0.011	0.025

### Comparison of HAMD scores

Differences of HAMD scores between two groups before treatment were not statistically significant. HAMD scores of observation group were lower than those of control group at the end of treatment and 8 weeks of follow-up (*P*<0.05) (Table 3).

**Table 3:** HAMD score comparison (points)

***Groups***	***Cases (n)***	***Before treatment***	***End of treatment***	***8 weeks of follow-up***
Observation	66	23.89±1.23	8.47±1.44	5.77±1.04
Control	66	24.51±1.35	13.51±1.65	8.91±1.87
*t*		1.806	2.412	2.823
*P*		0.078	0.030	0.005

### Comparison of clinical efficacy

The total clinical effective rates of the observation group and the control group were 92.43% and 86.37%, respectively, and the difference was statistically significant (*P*<0.05) (Table 4).

**Table 4:** Comparison of clinical effects [n (%)]

***Groups***	***Cases*** **(n)**	***Cured***	***Significant effective***	***Effective***	***Invalid***	***Total effective rate***
Observation	66	41 (62.14)	9 (13.63)	11 (16.66)	5 (7.57)	61 (92.43)
Control	66	26 (39.41)	18 (27.27)	13 (19.69)	9 (13.63)	57 (86.37)
*χ*^2^						7.158
*P*						0.019

## Discussion

In recent years, with the continuous development of rehabilitation medicine, patients with chronic limb pain now have been treated with comprehensive rehabilitation therapy such as exercise therapy, intermediate frequency pulse electrotherapy, traction, etc., which has improved the rate of improvement (6). At the same time, in the treatment of patients with chronic limb pain, some scholars also realize that patients’ anxiety, depression and other emotions will affect the pain. Appropriate health education and psychological care should be given to patients to improve their negative mood and ease work stress and life stress (7). Therefore, our study investigated the clinical efficacy of physical factors combined with early psychological intervention in treatment of patients with chronic limb pain. Our study provided guidance to the treatment of chronic limb pain.

Patients with chronic limb pain often present with neck, shoulder, back and leg pain, which are mostly caused by aseptic inflammation and chronic strain. Traditional Chinese medicine fumigation treatment used in the two groups in this study can stimulate the skin, promote telangiectasia, help lymphatic and blood circulation, and promote metabolism. Safflower, angelica, and pepper have the effect of relaxing the muscles, promoting blood circulation, relieving swelling and relieving pain. *Eucommia, aconite* and *Rhizoma cibotii* have the effect of warming the liver and kidney, and the sputum has the effect of reducing swelling and dampness (8, 9). Therefore, fumigation treatment of traditional Chinese medicine can effectively relieve the pain.

Through the impulse of audio waves to the brain’s nerve center, audio electrotherapy can protect the cerebral cortex, thus improving blood circulation, swelling, anti-inflammatory, analgesic, and phlegm, and effectively alleviating chronic limb pain (10). Patients with chronic limb pain are prone to negative psychological state under long-term pain, leading to some observable and obvious adverse physical reactions, which in turn aggravates the degree of negative psychology and causes serious sorrow, fear, depression, depression, anxiety, pain and other emotions to form a vicious circle. Pain can cause depression, and depression can aggravate pain (11). Antidepressants can effectively alleviate or even cure chronic pain. Results of this study showed that the PHQ-15 pain factor score, VAS score, and HAMD score of the observation group were lower than those of the control group at the end of treatment and at 8 weeks of follow-up. Total clinical effective rates of observation group and control group were 92.43% and 86.37%, respectively, and the difference was statistically significant. This is because that early psychological intervention through the relaxation training and abdominal breathing training can effectively help patients relax, thereby improving pain. Early psychological intervention can effectively improve the negative emotions of patients’ anxiety and depression, help patients to comprehend and eliminate misunderstandings by finding false cognitions that cause bad emotions, and then establish reasonable cognition (12).

Early psychological intervention can provide psychological support for patients, eliminate fear, relieve pain, reduce the use of analgesic drugs, enhance the patient’s sense of self-control of pain, and then improve recovery. In addition, there are many forms of psychological intervention, the operation is simple, and the cost is low.

## Conclusion

Patients with chronic limb pain combined with physical intervention with physical factors can significantly relieve pain and ease depression. It should be promoted in clinical practices.

## Ethical considerations

Ethical issues (Including plagiarism, informed consent, misconduct, data fabrication and/or falsification, double publication and/or submission, redundancy, etc.) have been completely observed by the authors.

## References

[1] Herin F Vezina M Thaon I Soulat JM Paris C ESTEV group (2014). Predictive risk factors for chronic regional and multisite musculoskeletal pain: a 5-year prospective study in a working population. Pain, 155(5): 937–943. 24561229 10.1016/j.pain.2014.01.033

[2] Thomas S (2015). Effectiveness of electromyographic biofeedback, mirror therapy, and tactile stimulation in decreasing chronic residual limb pain and phantom limb pain for a patient with a shoulder disarticulation. Journal of Prosthetics and Orthotics, 27(3): 68–76.

[3] Drummond PD Finch PM (2014). A disturbance in sensory processing on the affected side of the body increases limb pain in complex regional pain syndrome. Clin J Pain, 30(4): 301–306. 23792344 10.1097/AJP.0b013e31829ca4fc

[4] Deer TR Mekhail N Provenzano D (2014). The appropriate use of neurostimulation of the spinal cord and peripheral nervous system for the treatment of chronic pain and ischemic diseases: the Neuromodulation Appropriateness Consensus Committee. Neuromodulation, 17(6): 515–550; discussion 550. 25112889 10.1111/ner.12208

[5] van Hooff ML Spruit M O’Dowd JK van Lankveld W Fairbank JC van Limbeek J (2014). Predictive factors for successful clinical outcome 1 year after an intensive combined physical and psychological programme for chronic low back pain. Eur Spine J, 23(1): 102–112. 23771553 10.1007/s00586-013-2844-z PMC3897840

[6] McCormick Z Chang-Chien G Marshall B Huang M Harden RN (2014). Phantom limb pain: a systematic neuroanatomical-based review of pharmacologic treatment. Pain Med, 15(2): 292–305. 24224475 10.1111/pme.12283

[7] Zhang R Chomistek AK Dimitrakoff JD Giovannucci EL Willett WC Rosner BA Wu K (2015). Physical activity and chronic prostatitis/chronic pelvic pain syndrome. Med Sci Sports Exerc, 47(4): 757–764. 25116086 10.1249/MSS.0000000000000472 PMC4324388

[8] Wideman TH Finan PH Edwards RR Quartana PJ Buenaver LF Haythornthwaite JA Smith MT (2014). Increased sensitivity to physical activity among individuals with knee osteoarthritis: relation to pain outcomes, psychological factors, and responses to quantitative sensory testing. Pain, 155(4): 703–711. 24378879 10.1016/j.pain.2013.12.028

[9] Fuentes J Armijo-Olivo S Funabashi M Miciak M Dick B Warren S Rashiq S Magee DJ Gross DP (2013). Enhanced therapeutic alliance modulates pain intensity and muscle pain sensitivity in patients with chronic low back pain: an experimental controlled study. Physical Therapy, 94(4): 477–489. 24309616 10.2522/ptj.20130118

[10] Elander J (2014). A review of evidence about behavioural and psychological aspects of chronic joint pain among people with haemophilia. Haemophilia, 20(2): 168–175. 24533948 10.1111/hae.12291

[11] Baumeister H Seifferth H Lin J Nowoczin L Luking M Ebert D (2015). Impact of an Acceptance Facilitating Intervention on Patients’ Acceptance of Internet-based Pain Interventions: A Randomized Controlled Trial. Clin J Pain, 31(6): 528–535. 24866854 10.1097/AJP.0000000000000118

[12] Trompetter HR Bohlmeijer ET Veehof MM Schreurs KM (2015). Internet-based guided self-help intervention for chronic pain based on Acceptance and Commitment Therapy: a randomized controlled trial. J Behav Med, 38(1): 66–80. 24923259 10.1007/s10865-014-9579-0

